# A complete mitochondrial genome sequence of Ogura-type male-sterile cytoplasm and its comparative analysis with that of normal cytoplasm in radish (*Raphanus sativus* L.)

**DOI:** 10.1186/1471-2164-13-352

**Published:** 2012-07-31

**Authors:** Yoshiyuki Tanaka, Mizue Tsuda, Keita Yasumoto, Hiroshi Yamagishi, Toru Terachi

**Affiliations:** 1 31 Laboratory, Faculty of Life Sciences, Kyoto Sangyo University, Motoyama, Kamigamo, Kita-ku, Kyoto, 603-8555, Japan

## Abstract

**Background:**

Plant mitochondrial genome has unique features such as large size, frequent recombination and incorporation of foreign DNA. Cytoplasmic male sterility (CMS) is caused by rearrangement of the mitochondrial genome, and a novel chimeric open reading frame (ORF) created by shuffling of endogenous sequences is often responsible for CMS. The Ogura-type male-sterile cytoplasm is one of the most extensively studied cytoplasms in *Brassicaceae*. Although the gene *orf138* has been isolated as a determinant of Ogura-type CMS, no homologous sequence to *orf138* has been found in public databases. Therefore, how *orf138* sequence was created is a mystery. In this study, we determined the complete nucleotide sequence of two radish mitochondrial genomes, namely, Ogura- and normal-type genomes, and analyzed them to reveal the origin of the gene *orf138*.

**Results:**

Ogura- and normal-type mitochondrial genomes were assembled to 258,426-bp and 244,036-bp circular sequences, respectively. Normal-type mitochondrial genome contained 33 protein-coding and three rRNA genes, which are well conserved with the reported mitochondrial genome of rapeseed. Ogura-type genomes contained same genes and additional *atp9*. As for tRNA, normal-type contained 17 tRNAs, while Ogura-type contained 17 tRNAs and one additional *trnfM*. The gene *orf138* was specific to Ogura-type mitochondrial genome, and no sequence homologous to it was found in normal-type genome. Comparative analysis of the two genomes revealed that radish mitochondrial genome consists of 11 syntenic regions (length >3 kb, similarity >99.9%). It was shown that short repeats and overlapped repeats present in the edge of syntenic regions were involved in recombination events during evolution to interconvert two types of mitochondrial genome. Ogura-type mitochondrial genome has four unique regions (2,803 bp, 1,601 bp, 451 bp and 15,255 bp in size) that are non-syntenic to normal-type genome, and the gene *orf138* was found to be located at the edge of the largest unique region. Blast analysis performed to assign the unique regions showed that about 80% of the region was covered by short homologous sequences to the mitochondrial sequences of normal-type radish or other reported *Brassicaceae* species, although no homology was found for the remaining 20% of sequences.

**Conclusions:**

Ogura-type mitochondrial genome was highly rearranged compared with the normal-type genome by recombination through one large repeat and multiple short repeats. The rearrangement has produced four unique regions in Ogura-type mitochondrial genome, and most of the unique regions are composed of known *Brassicaceae* mitochondrial sequences. This suggests that the regions unique to the Ogura-type genome were generated by integration and shuffling of pre-existing mitochondrial sequences during the evolution of *Brassicaceae*, and novel genes such as *orf138* could have been created by the shuffling process of mitochondrial genome.

## Background

Compared with animal mitochondrial genomes that are compact and relatively uniform in size, plant mitochondrial genomes are extremely complex and have characteristic features, including large genome size, frequent reorganization and incorporation of foreign DNA [1]. Plant mitochondrial genome varies in size from 208 kb in *Brassica hirta* to 11.3 Mb in *Silene conica*[2,3]*,* due to the expansion of non-coding sequence and duplication of a large segment. It is also well known that plant mitochondrial genome evolves rapidly in structure and slowly in sequence. Plant mitochondria contain a number of repeats. The recombination via repeat sequences is believed to be responsible for extensive structural change. Incorporation of foreign DNA fragments that originated from plastid and nuclear genomes also can be found in plant mitochondrial genome. Because of frequent recombination and incorporation of foreign DNA, extensive genome reorganization and gene-order shuffling may occur. These changes in mitochondrial genome can produce novel open reading frames, some of which result in cytoplasmic male sterility (CMS) [4].

CMS is a maternally inherited trait in which a plant is unable to produce functional pollen. CMS is an economically important trait for F_1_ hybrid seed production in many crops. CMS has been widely observed in higher plants, and many reports show that CMS is caused by alteration of the mitochondrial genome [5]. These alterations often create a novel open reading frame (ORF), which consists of a chimeric sequence generated by gene shuffling or by fusion between a native gene(s) and/or an unknown sequence(s).

The Ogura male-sterile cytoplasm, which was originally found in an unknown variety of Japanese radish, is the most widely studied cytoplasm in *Brassicaceae*[6]. This cytoplasm is used in F_1_ seed production of European, Chinese and Japanese radishes. The cytoplasm was introduced into *Brassica* crops by intergeneric hybridization and somatic cell fusion, and has been utilized for F_1_ seed production in *Brassica* crops such as rapeseed worldwide [7,8].

A mitochondrial gene, *orf138*, is responsible for Ogura male sterility and specifically present in the mitochondrial genome of various radishes with Ogura-type cytoplasm [9]. The accumulation of ORF138 protein is observed in plants expressing the CMS phenotype, and the association of ORF138 with the inner mitochondrial membrane of male-sterile plants was reported [10]. Although it appeared that expression of the nuclear chalcone synthase gene, which is related to flavonoid biosynthesis, is inhibited in the anther of Ogura radish [11], the molecular mechanism underlying Ogura CMS by ORF138 protein is not known. The sequence of the gene *orf138* itself is also interesting; there is no evidence that *orf138* is a chimeric gene consisting of native mitochondrial genes or sequence. No sequence homologous to *orf138* is found in public databases; therefore, the origin of *orf138* is a mystery. In addition, it has been shown that the mitochondrial genome of Ogura-type cytoplasm is highly rearranged compared with that of normal cytoplasm in radish. These rearrangements occurred in the loci *atp1atp6cox1* and most notably *orf138-atp8*[12-17]. The gene *orf138* is specifically located in the 5’ upstream region of the gene *atp8* in Ogura radish, whereas the region is occupied by the gene *cox1* in normal radish [16]. In the evolution of *Raphanus,* how and when these extensive rearrangements occurred between two types of mitochondrial genome are not known.

In this study, in order to obtain a better understanding of the origin of *orf138* and Ogura-type mitochondrial genome in radish, we determined the complete nucleotide sequences of two mitochondrial genomes of radish, that is, Ogura- and normal-type mitochondrial genomes, and compared their structures. Gene contents and unique regions for each genome were analyzed in detail at the sequence level. Furthermore, we focused on short repeated sequences that may be involved in the recombination, leading to extensive rearrangements of the two genomes and to generation of the gene *orf138*.

## Results

### Radish mitochondrial genome

**Figure 1:**
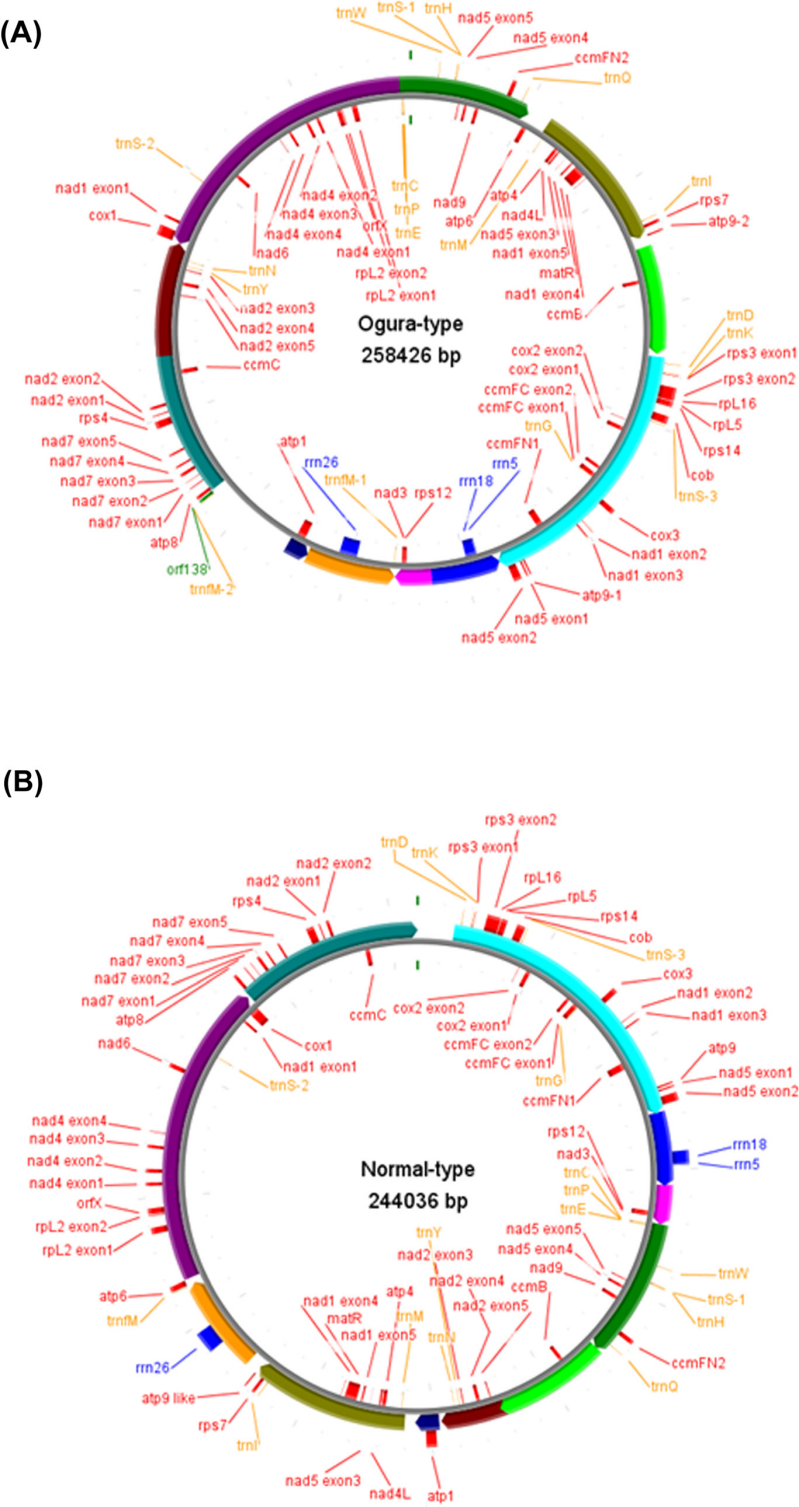
**The organization of Ogura-type (A) and normal-type (B) mitochondrial genomes represented as a "master circle".** Features on forward and reverse strands are drawn on the outside and inside of the circles, respectively. Protein-coding genes are shown in red, rRNAs in blue, tRNAs in orange and *orf138* in lime green. The arcs in the same colors indicate syntenic regions between Ogura- and normal-type genomes (refer to Figure 2). Genome maps were made with CGviewer [49].

The Ogura and normal mitochondrial genomes are assembled as 258,426-bp and 244,036-bp circular molecules, respectively (Figure 1). The overall GC content of both mtDNAs is 45.2%, being comparable to those of other mtDNAs of *Brassicaceae*. About 7% of Ogura and 3% of normal sequences are unique to each mitochondrial genome, and 80% of Ogura and 84% of normal sequences show high similarity to the sequence of rapeseed (*Brassica napus* L.) mitochondrial genome (GenBank: AP006444.1) [18].

### Gene organization of radish mitochondrial genome

Normal-type mitochondrial genome contained 33 protein-coding and three rRNA genes, while Ogura-type genomes contained same genes and one additional *atp9* (*atp9-2*). Gene content is identically conserved between the two radish mitochondrial genomes and it is also identical to that of rapeseed, except *atp9-2* in Ogura-type genome. Normal-type genome contains similar sequence to *atp9-2,* but 3’ part of this sequence lacks similarity to *atp9-2*. The *atp9-2* like sequence in normal-type genome encodes 70 amino acid protein, which is lacking *atp9* domain partially. Eighteen of the conserved 33 protein-coding genes produce components of the electron transport chain and ATP synthase: nine subunits of complex I(*nad1nad2nad3nad4nad4Lnad5nad6nad7* and *nad9*), apocytochrome b (*cob*) of complex III, three subunits of complex IV (*cox1cox2* and *cox3*) and five subunits of complex V (*atp1atp4atp6atp8* and *atp9*). Five additional proteins are involved in the biogenesis of cytochrome c (*ccmBccmCccmFN1*/*ccmFN2* and *ccmFC*). Another eight genes encode ribosomal proteins (*rpl2rpl5* and *rpl16*, and *rps3rps4rps7rps12* and *rps14*). The two remaining genes encode maturase (*matR*) and *orfX*. The gene *ccmFN* is divided into two reading frames in mitochondrial genomes of radish like in those of rapeseed and *Arabidopsis*[18,19]. Three rRNA genes (*rrn5rrn18* and *rrn26*) are highly conserved between the two mitochondrial genomes of radish.

The 5’ region of the gene *atp6* is highly diverged between the two radishes because of recombination that occurred in the locus [20]. The 3’ end of Ogura-type *orfX* also lacks similarity to that of normal-type. This is caused by a 48-bp repeated sequence that is regarded as a minisatellite. Eleven SNPs were identified in the nine genes: four were synonymous and seven were non-synonymous (Table 1). As for tRNA, both Ogura and normal mitochondrial genomes contain 17 tRNAs, whereas Ogura genome has an additional *trnfM* (*trnfM-2*). The *trnfM-2,* which is located close to the gene *orf138* (Figure 1)*,* has a specific SNP compared with other sequenced mitochondrial *trnfMs* in *Brassicaceae*.

**Table 1:** Difference in genes coding protein between normal-type and Ogura-type

**Gene**	**Nucleic acid**	**Amino acid**^**b**^
*nad4*	1010 T-C	Non-synonymous (337 L-P)
*cox1*	117 G-T	Synonymous
*atp6*	no homology in 5′ terminal	
*atp8*	370 A-C	Non-synonymous (124 I-L)
	444 T-C	Non-synonymous (150 V-A)
*atp9*	65 A-G	Non-synonymous (22 I-V)
*rpL2*	840 C-T	Synonymous
*rpL16*	492 G-A	Synonymous
*rps4*	776 C-T	Non-synonymous (259 S-F)
*ccmC*	351 G-A	Synonymous
*ccmFC*	605 A-G	Non-synonymous(202 E-G)
	606 G-T	
*orfX*	no homology in 3′ terminal	

a Location of SNP (Normal-Ogura).

b Location of amino acid mutation (Normal-Ogura).

We searched for a sequence homologous to the *orf138* in Ogura and normal mtDNA sequences. Only a 67-bp sequence, which includes the 3’ terminal of the *orf138* sequence and its 3’ UTR, was detected. This sequence is homologous to 5’ flanking region of *ccmFN1* in reported *Brassicaceae* genomes. However, except for this short sequence, no homology was found between the main part of the gene *orf138* and other parts of the *Brassicaceae* mitochondrial genome.

### Reorganization between two types of radish mitochondrial genome

**Figure 2:**
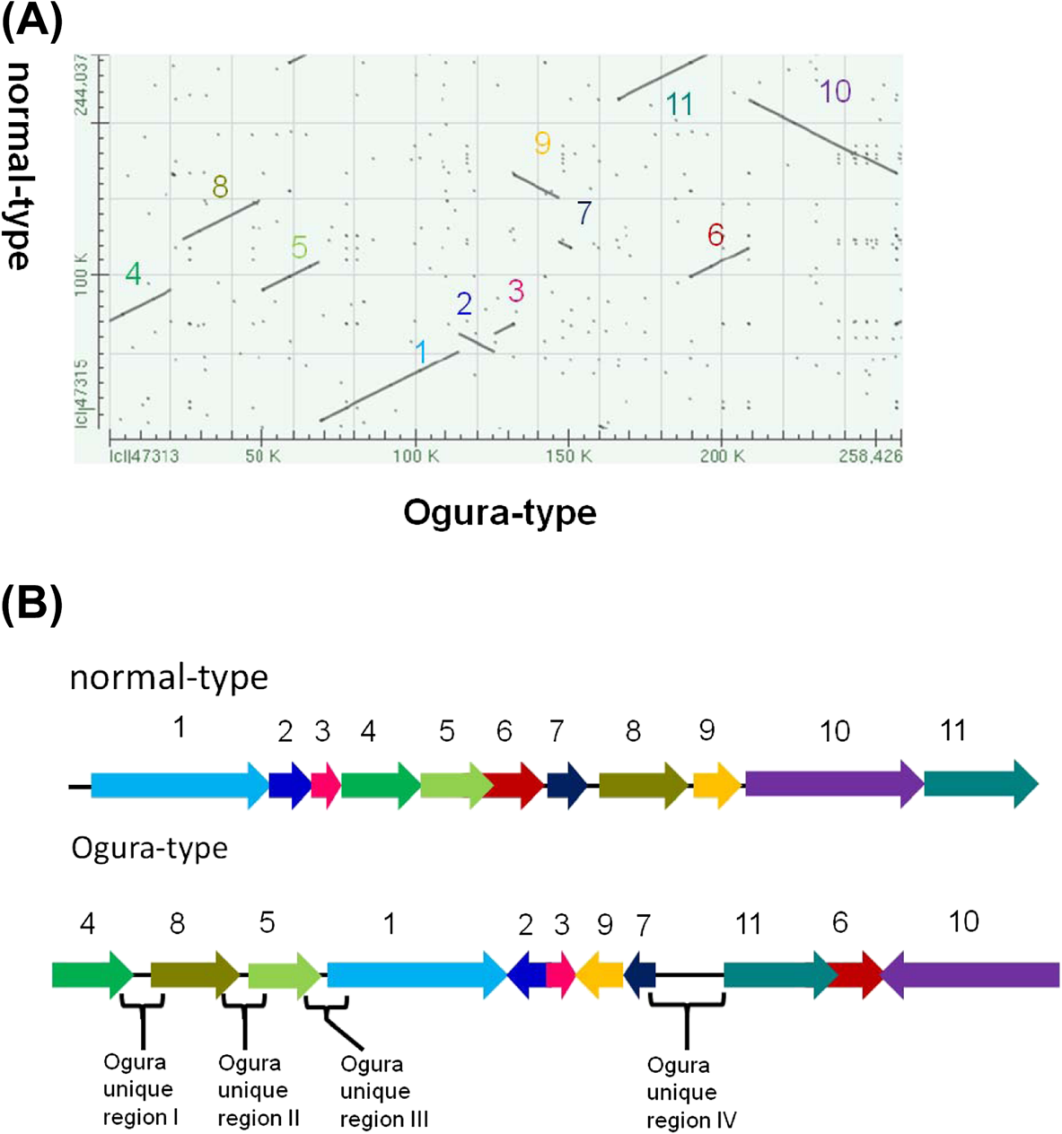
**Comparison of the location and orientation of syntenic regions between Ogura- and normal-type mitochondrial genomes.** (**A**) Ogura-type genome on the X-axis, plotted against normal-type genome on the Y-axis. The numbers for the syntenic regions correspond to those indicated in (**B**). (**B**) Schematic illustration of the eleven syntenic regions in radish mitochondrial genomes.

The bl2seq analysis suggested that the radish mitochondrial genome consists of 11 syntenic regions (length >3 kb) (Figure 2). The sequence in the Ogura-type mitochondrial genome shows quite high homology (99.9%) to that of normal mitochondrial genome within these syntenic regions. This analysis revealed that Ogura-type mitochondrial genome has a large unique region (15,255 bp) between syntenic regions 7 and 11. This unique region corresponds to the “region A” that was reported by Makaroff et al. [12]. The gene *orf138* is located at one of the terminals of this region (Figure 1).

**Figure 3:**
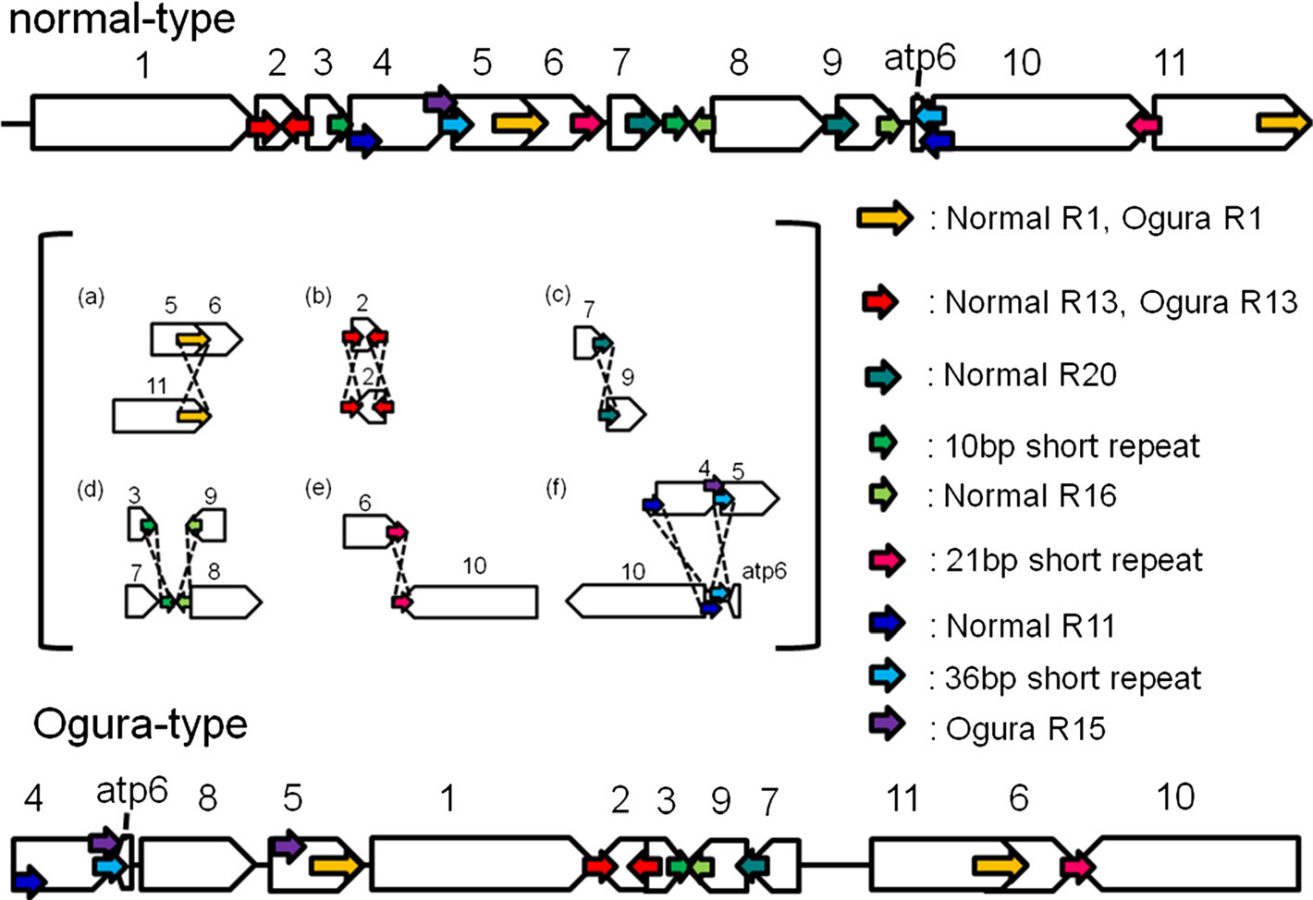
**The possible rearrangements that had occurred between two Ogura- and normal-type genomes via short repeated sequences.** Large blank arrows indicate each syntenic region. Small arrows in the same color show the position and orientation of repeats. The length and location of the repeats are summarized in Additional file 1 and Additional file 2.

Repeats in two mitochondrial genomes have been investigated. If a pair of sequences has over 90% similarity, the pair is defined as a repeat. A pair of large repeats of 9,732 bp was found in Ogura-type mitochondrial genome, while a pair of large repeats of 5,530 bp was identified in normal-type. Even though the length of large repeats is different, sequence of the large repeats are highly conserved within 5,530 bp. In Ogura-type mitochondrial genome, the large repeats are located in syntenic regions 5 and 11/6 (Figure 3, Ogura R1). A 9,732-bp sequence, corresponding to Ogura-type repeat, is conserved in syntenic region 5/6 of the normal-type genome, but it is truncated in syntenic region 11 because normal-type genome has a specific sequence between syntenic regions 1 and 11. In rapeseed mitochondrial genome, a 2,427-bp sequence that includes the gene *cox2* was reported as a large direct repeat. Large repeated sequences found in *Raphanus* are completely different from those reported in rapeseed [18]. These large repeats are considered to be involved in the formation of a tripartite structure; each radish mitochondrial genome could recombine into two sub-genomic circles. Two expected sub-genomic circles are 130,760 bp and 127,666 bp in Ogura-type, while they are 139,398 bp and 104,638 bp in normal-type. In addition, these repeats are also related to reorganization among syntenic regions 5, 6 and 11 (Figure 3a).

We defined 22 and 23 short repeats in Ogura- and normal-type genomes, respectively (Additional file 1 and Additional file 2). These repeats can account for reorganization of mitochondrial genome via homologous recombination. We have identified repeats that can be involved in reorganization from normal- to Ogura-type mitochondrial genomes, although the direction of changes cannot be fixed. The pair of inverted repeats (Normal R13) is located on either side of syntenic region 2 in normal-type genome. These inverted repeats may change the orientation of syntenic region 2 in Ogura-type genome (Figure 3b). In normal-type mitochondrial genome, a pair of direct repeats (Normal R20) is located at the edge of syntenic regions 7 and 9. These repeats can be related to generation of a linkage between 7 and 9 in Ogura-type genome (Figure 3c). In normal-type genome, Normal R16 is present at the edge of syntenic region 9 and in the unique sequence located between syntenic regions 7 and 8 in the inverted orientation. A 10-bp short repeat is also present in this unique sequence, and it exists at the edge of syntenic region 3 in the direct orientation. These repeats can be associated with the generation of a linkage between syntenic regions 3 and 9 in Ogura-type genome (Figure 3d). Syntenic regions 6 and 10 in normal-type genome have a 21-bp short inverted repeat. This inverted repeat can be involved in inversion and recombination between syntenic regions 6 and 10 in Ogura-type genome (Figure 3e). Finally, in normal-type genome, syntenic region 4 has Normal R11 and a short 36-bp repeat at each edge. Normal R11 is also present at one edge of syntenic region 10, being overlapped with a 36-bp repeat. In Ogura-type genome, a linkage between syntenic regions 10 and 4 is generated via these two sequences. This recombination event also changes the location of the gene *atp6* (Figure 3f). Moreover, in Ogura-type genome, two copies of a short direct repeat (Ogura R15) are present at the edge of syntenic regions 4 and 5, between which syntenic region 8 is inserted with a novel sequence (Figure 3f).

### Open reading frames that are unique to each mitochondrial genome type

A comprehensive comparison of predicted ORFs was conducted between Ogura- and normal-type mitochondrial genomes. We picked up unique ORFs that can encode over 100 amino acids and were generated by recombination. As a result, six ORFs including *orf138* were specifically detected in Ogura-type mitochondrial genome (Table 2). The five Ogura-type ORFs were located between syntenic regions 7 and 11, whereas *orf344* was in syntenic region 3. With the exception of *orf138,* the nucleotide sequences of the five ORFs showed high similarity to the reported sequences in plant mitochondrial genomes of other species. As for the normal-type genome, three unique ORFs were detected. The *orf322* has strong similarity to the *orf344* in Ogura-type. The extension of *orf344* was caused by a new initiation codon that was created in 5’ region of *orf322* through a recombination between syntenic regions 3 and 9. The two remaining ORFs were located within the region unique to the normal-type genome located between syntenic regions 11 and 1. Protein Blast analysis was conducted to characterize unique ORFs. Even though homologous amino acid sequences were not found for *orf150* in normal-type and for *orf122* and *orf138* in Ogura-type, other unique ORFs encoded similar proteins that have been reported in the mitochondrial genome of rapeseed or *Arabidopsis*.

**Table 2:** Mitotype specific open-reading frames caused by recombination

	**ORF**	**Similarity of predicted protein**	**Location**
Ogura-type specific	*orf122*	No similarity	between syntenic regions 7 and 11
	*orf154*	AAG51754.1 reverse transcriptase, putative[*Arabidopsis thaliana*]	between syntenic regions 7 and 11
	*orf117*	YP_717156.1 hypothetical protein BrnapMp059 [*Brassica napus*]	between syntenic regions 7 and 11
	*orf138*	No similarity	between syntenic regions 7 and 11
	*orf102*	NP_085574.1 hypothetical protein ArthMp044 [*Arabidopsis thaliana*]	between syntenic regions 7 and 11
	*orf344*	YP_717145.1 hypothetical protein BrnapMp048 [*Brassica napus*]	the edge of syntenic region 3
normal-type specific			
	*orf322*	YP_717145.1 hypothetical protein BrnapMp048 [*Brassica napus*]	the edge of syntenic region 3
	*orf150*	No similarity	between syntenic regions 1 and 10
	*orf145*	NP_085575.1 hypothetical protein ArthMp101 [*Arabidopsis thaliana*]	between syntenic regions 1 and 10

### Regions unique to the Ogura-type mitochondrial genome

Ogura-type mitochondrial genome has four unique regions that are non-syntenic to normal-type mitochondrial genome. Unique region I is 2,802 bp, which is located between syntenic regions 4 and 8 (Figure 2B). Unique regions II and III are 1,601-bp and 451-bp sequences that are present between syntenic regions 8 and 5, and between syntenic regions 5 and 1, respectively. The largest unique region IV is located between syntenic regions 7 and 11 (Figure 2B). Total length of the unique regions in Ogura-type genome is 20,110 bp, which occupies 7% of the whole Ogura-type mitochondrial genome. The largest unique region contains five unique ORFs including *orf138*.

**Figure 4:**
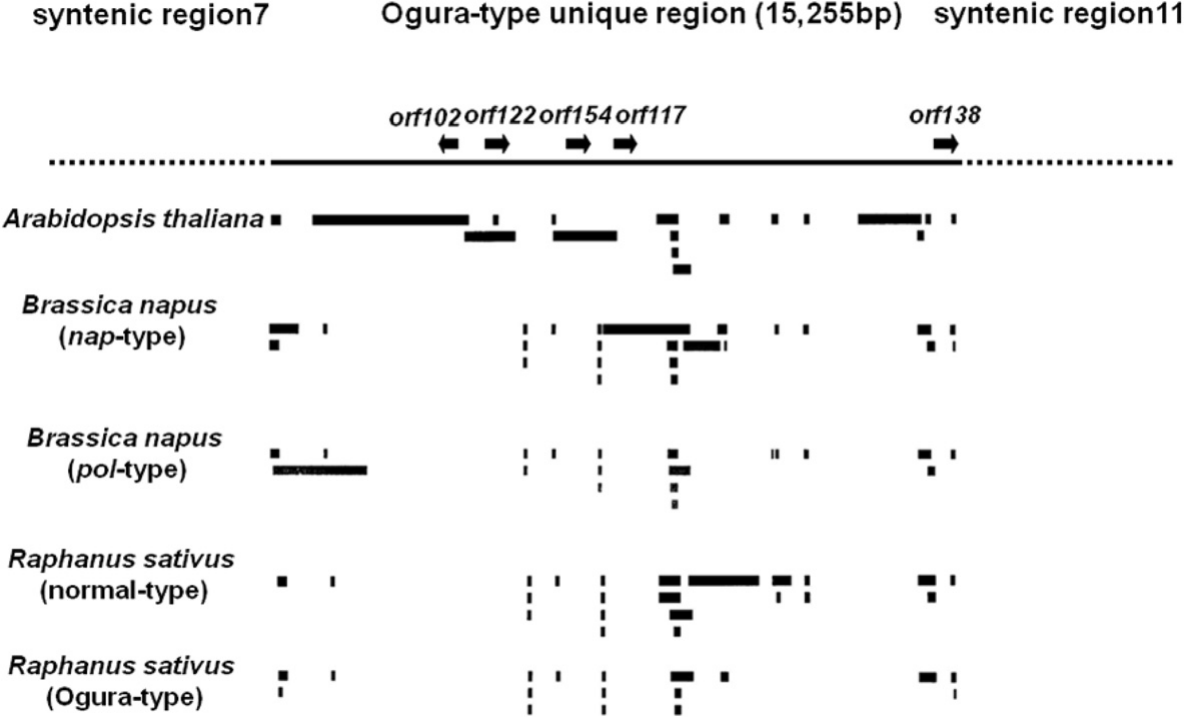
**The sequences homologous to *Brassicaceae* mitochondrial genome plotted on Ogura-type specific region IV.** A black line indicates the region specific to the Ogura-type mitochondrial genome (15,255 bp in size) located between syntenic regions 7 and 11. The gene *orf138* is located at the edge of this region. Black boxes indicate the sequence homologous to radish and/or other *Brassicaceae* mitochondrial genomes.

To obtain more information on the sequence unique to Ogura-type genome, nucleotide Blast search was conducted to assign the sequences. The search showed that over 80% of these regions consist of sequences homologous to those reported for *Brassicaceae* or radish mitochondrial genomes. Bl2seq analyses were further conducted between the sequence unique to Ogura-type and each of five mitochondrial genomes in *Brassicaceae* (normal and Ogura-type radish, *A. thaliana* and two types of *Brassica napus*). Seventy-two percent of unique region I (2,044 bp in a total of 2,802 bp) and 100% of unique region II (1,601 bp) were occupied by five mitochondrial sequences of *Brassicaceae* (Additional file 3). As for the smallest unique region III, 97% of the sequence (440 bp in a total of 451 bp) was shown to have similarity to rapeseed mtDNAs. As for the largest unique region IV, sequences homologous to the five *Brassicaceae* mitochondrial genomes covered 80% of this region (12,187 bp in a total of 15,255 bp), even though *orf138* sequence itself had no similarity. Homologous sequences detected by bl2seq were plotted on unique region IV in Ogura-type mitochondrial genome (Figure 4). Homologous sequences were scattered throughout this region. Insertion of plastid or nuclear sequence has not been detected in this region. It seems, therefore, that the region unique to Ogura-type mitochondrial genome was created by frequent recombination and shuffling of mitochondrial DNA sequences during the evolution of *Brassicaceae*.

## Discussion

### Comparative sequence analysis of Ogura- and normal-type mitochondrial genomes

Ogura-type mitochondrial genome was 14 kb larger than that of normal type. Region IV that is unique to Ogura-type mitochondrial genome mainly contributes to this size difference. Comparative analysis has been performed between mitochondrial genomes of CMS and normal lines in other higher plant species. In sugar beet, Owen-type CMS line has a significantly larger mitochondrial genome than fertile line because the Owen-type mitochondrial genome contains one 86-kb large repeat [21]. In wheat, the mitochondrial genome of the CMS line Ks3 was reported to be 192 kb larger than that of common wheat [22]. This difference is also caused by additional large repeats, including a 98-kb repeat. In contrast, a rice (*Oryza sativa*) LD-CMS line has a smaller mitochondrial genome than fertile line, while CW-CMS derived from wild rice (*O. rufipogon*) has a larger genome than fertile line [23]. The difference in mitochondrial genome size within the *Oryza* genus is also attributable to large repeats. It should be noted that the size difference between two radish mitochondrial genomes mainly arises from a novel sequence of 15,255 bp, not from a large repeated sequence.

The reported *Brassicaceae* species including *Brassica* and *Arabidopsis* have small mitochondrial genomes among higher plants [24]. It is also known that *Brassicaceae* mitochondrial genomes reported so far do not contain much nuclear sequence. The number of large repeats (>1 kb) is less in reported *Brassicaceae* (*Arabidopsis* and *Brassica*) than in other plants species [25]. While mitochondrial genomes of rice or wheat have more than ten large repeats, *Brassica* and *Raphanus* have only two large repeats. In addition, sequences derived from nuclear or plastid genomes were rarely observed. This is one of the reasons why mitochondrial genomes of *Brassica* and *Raphanus* are small. Because of the small genome size and few large repeats, we can consider *Brassica* and *Raphanus* as species that have the “simple” mitochondrial genomes in higher plants. An understanding of the remarkable reorganization events that occurred in the simple radish genome will provide us with a clear picture of mitochondrial genome evolution, such as insertion of a 15-kb large novel sequence in Ogura-type.

As expected, gene contents and their sequences were highly conserved between the two mitochondrial genomes in radish. The 5’ sequence of the *atp6* in Ogura-type mitochondrial genome is, however, different from that of normal-type. Recombination had occurred at the site of syntenic region 4 and *atp6*, which produced a new initiation codon for *atp6* in Ogura-type genome. According to the genome sequence, Ogura-type *atp6* encodes a different polypeptide from that of normal-type *atp6*. However, the N-terminal of ATP6 polypeptide of Ogura-type is processed to produce a protein identical to normal-type ATP6 [20]. The 3’ end of *orfX* also lacks similarity to the sequence of normal-type. This is caused by a 48-bp repeated sequence regarded as a minisatellite. This minisatellite will be a useful marker to detect polymorphism among radish mitochondrial genomes including Ogura and normal types [26]. A total of 11 SNPs were detected in nine protein-coding genes between Ogura and normal types. The number of SNPs in radish was similar to that in sugar beet; the sugar beet Owen-CMS line contains 24 SNPs in 11 protein-coding genes compared with the fertile lines [21]. As for tRNA, 17 tRNAs were highly conserved between two radish mitochondrial genome types, but Ogura-type contains one additional *trnfM* (*trnfM-2*). This additional *trnfM-2* is located near *orf138*, and it is interesting to note that this *trnfM-2* has a unique SNP that has not been found in other reported *trnfMs* in *Brassicaceae*. As for unique ORFs, Ogura-type has six unique ORFs including *orf138*. These ORFs can be related to unique biological characteristics in Ogura-type cytoplasm. In particular, transcription of four ORFs (*orf122orf154orf117* and *orf102*) that are specific to Ogura-type genome should be investigated in future study.

### The origin of Ogura-type unique sequence

CMS-related ORFs have been isolated in various plant species, such as *T-urf13* in maize [27], *orf522* in sunflower [28], *pcf* in petunia [29], *orf456* in pepper [30], *orf107* in sorghum [31] and *orf79* in rice [32]. In *Brassicaceae* crops, in addition to *orf138* in radish, three CMS-related ORFs have been reported: *orf224* and *orf222* in rapeseed [33], and *orf267* in *Brassica tournefortii*[34]. With the exception of *orf138*, all these CMS-related ORFs have a chimeric structure that contains the sequence of known mitochondrial genes. The *orf138*, however, does not contain any conserved mitochondrial sequence. The sequence of *orf138* is totally novel, and is not present in normal-type mitochondrial genome of radish. It is quite interesting to reveal how the *orf138* sequence was created during the evolution of radish mitochondrial genome. However, it now appears difficult to infer the origin of the gene *orf138*, since no sequence homologous to the main part of *orf138* is present in either Ogura- or normal-type mitochondrial genome.

In this study, we have determined the nucleotide sequence for unique region IV (15,255 bp in size) in Ogura-type mitochondrial genome. The gene *orf138* is located in this region. Blast analysis using the sequence in region IV as a query showed that 80% of this region has similarity to the mitochondrial sequences in other *Brassicaceae* plants, including rapeseed and *Arabidopsis*. The analysis also showed that the homologous sequences are scattered throughout unique region IV (Figure 4). Therefore, region IV is a mosaic of mitochondrial sequences from *Brassicaceae*, suggesting that the sequence was generated through shuffling and fusion of pre-existing mitochondrial sequences in ancestral *Brassicaceae*. The sequence of which the gene *orf138* consists may be one of the products of this shuffling process. It is unlikely that the sequence of *orf138* is derived from foreign DNA, such as a nuclear or plastid sequence, because no sequence with clear homology to foreign DNA is found within this unique region. However the possibility remains that they may be derived from horizontal gene transfer from unidentified organism.

This composition of novel sequence in radish contrasts to that reported for sugar beet. In sugar beet mitochondrial genome, 7.6% of the unique regions showed significant homology to previously determined mitochondrial sequences, 17.9% to nuclear DNA, 4.6% to mitochondrial episome and 0.1% to plastid DNA [35].

### The emergence of Ogura-type cytoplasm

**Figure 5:**
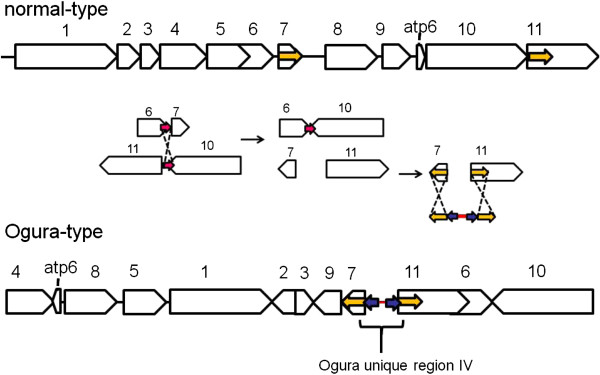
**A possible model for integration of the sequence (unique region IV) in Ogura-type mitochondrial genome.** Yellow small arrows indicate identical 176-bp inverted repeats at the edges of regions 7 and 11. On the insides of these repeated sequences, another pair of 28-bp inverted repeats is present (purple small arrows). Unique region IV had been integrated into radish mitochondria genome via these repeats when recombination between syntenic regions 7 and 11 occurred.

The sequence of Ogura-type unique region IV can be derived from a mixture of sequences in the reported *Brassicaceae* mitochondrial genome. The next question relates to how this unique region IV was generated or integrated into the radish mitochondrial genome. We searched for repeated sequences around this region. Ogura-type unique region IV was shown to be sandwiched between syntenic regions 7 and 11 (Figure 5). There were identical 176-bp inverted repeats at the edges of regions 7 and 11. On the insides of these repeated sequences, another pair of 28-bp repeated sequences was found in inverted orientation. These 28-bp sequences are specific to the Ogura-type mitochondrial genome, and not found in normal-type genome. This characteristic arrangement of repeats indicates that unique region IV was integrated into the radish mitochondrial genome when recombination between syntenic regions 7 and 11 occurred.

**Figure 6:**
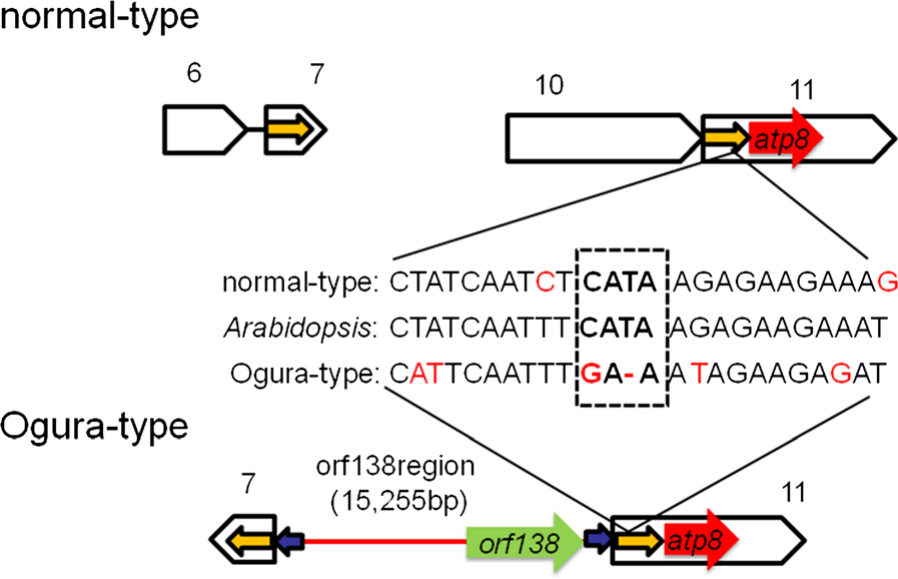
**An *atp8* promoter sequence affected by rearrangement among syntenic regions.** Alignment of nucleotide sequences shows the comparison of predicted promoters for the gene *atp8* among three mitochondrial genomes including *Arabidopsis*, and Ogura and normal radish. In Ogura-type genome, a promoter core (in a dotted box) is lost. This nucleotide change can affect transcription of the gene *atp8*.

Previously, we conducted sequence analysis on the *atp8* locus in normal- and Ogura-type cytoplasm from wild and cultivated radishes [36]. While plants with normal-type cytoplasm contained several types of sequence, plants with Ogura-type cytoplasm had only one type of sequence. These results suggested that a mutational event, which created linkage of *atp8* and *orf138*, occurred only once in the history of radish [36]. The present data indicate that this event probably occurred at the same time as when recombination between syntenic regions 7 and 11 occurred via 176-bp repeats and the unique region IV was integrated into the Ogura-type mitochondrial genome (Figure 5). Interestingly, the radish mitochondrial genome may have lost one putative promoter region for *atp8* by this recombination (Figure 6). In *Arabidopsis*, the promoters of mitochondrial genes have been determined [37]. A promoter sequence of *atp8* (CTATCAATCTCATAAGAGAAGAAAT) is almost conserved at the edge of syntenic region 11 of normal radish. The position of the promoter exactly matched the size of *atp8* transcript (ca. 600 bp in size) [16]. The recombination of syntenic regions 7 and 11 would destroy the promoter sequence. Therefore, recombination between syntenic regions 7 and 11 in normal radish can lead to loss of biological function in mitochondria. A reasonable hypothesis to explain the generation of Ogura-type mitochondrial genome is that the mitochondrial genome rearrangement that could compensate for the loss of biological function had been selected among many rearranged genomes. Integration of unique region IV can provide a new promoter for the gene *atp8*. Actually, in Ogura-type genome, *atp8* is co-transcribed with *orf138* and *trnfM*, while in normal-type, *atp8* is transcribed from its own promoter.

Another possibility to explain the emergence of Ogura-type mitochondrial genome is substoichiometric shift (SSS). SSS is a phenomenon by which copy number of the substoichiometric molecule dramatically increases over generations [38]. Research in some plants has shown that SSS is related to the occurrence of CMS [39,40]. Recently, it has been reported that two mitochondrial genome types, *pol* -type and *nap*-type, co-exist among many rapeseed cultivars, and SSS can explain the emergence of these CMS [41]. In radish with normal-type cytoplasm, *orf138* was reported to be present in substoichiometric molecules with low copy number by genomic PCR [42]. However, our previous analyses failed to detect the gene *orf138* in various normal radishes. The fact that no sequence homologous to *orf138* was found in the current pyrosequencing data (20,646,046 bp in total) from a normal radish suggests that substoichiometric molecules containing *orf138* are absent in normal radish. The discrepancy between our results and a previous study [42] can be attributed to the differences of materials used and to the conditions of PCR including primers, type of DNA polymerase and particularly amplification cycles (30 cycles [43,44] vs. 40 cycles in ref. [42]). However, our PCR conditions can amplify two different configurations of *orf138* regarded as subliminal fragments in various radishes with Ogura-type cytoplasm. This indicates that, if there were *orf138* sequences in normal radish, they would be at quite a low level, and the contribution of SSS as a possible mechanism to explain generation of the Ogura-type mitochondrial genome from normal-type genome remains to be elucidated.

Previously, we conducted a large-scale sequence analysis of *orf138* in 107 Japanese wild radishes, 29 cultivated radishes and seven *R. raphanistrum*[43]. On the basis of the pattern of mutation and the distribution of *orf138* variants, we inferred the mechanism behind the differentiation of Ogura-type cytoplasm. These studies showed that the original Ogura-type radish is derived from Japanese wild radish and that *orf138* sequence of Japanese wild radish was introduced from *R. raphanistrum*[44-46]. However, it was still unclear when Ogura-type cytoplasm was initially generated. This is still an open question, and comparative sequence analysis among more mitochondrial genomes in *Raphanus* and molecular evolutionary studies of the data should provide clues to resolve this issue.

## Conclusions

The complete nucleotide sequence of the mitochondrial genome from Ogura male-sterile radish reveals that the genome has been highly rearranged compared with that of normal radish. The radish mitochondrial genome consists of 11 syntenic regions. The reorganization of the genome occurred via a pair of large repeats and multiple pairs of short repeats, and produced four regions unique to the Ogura-type mitochondrial genome. Most of these unique regions consist of sequences homologous to known *Brassicaceae* mitochondrial genomes. Insertions of sequences derived from plastid or nuclear genome were not identified. This suggested that the unique regions were generated by integration and shuffling of pre-existing mitochondrial sequences during the evolution of *Brassicaceae*, and novel genes such as *orf138* could have been created by the shuffling process.

## Methods

### Plant materials

A cultivar 'Uchiki-Gensuke' (UC-G) and its male-sterile line 'MS-Gensuke' (MS-G) were used in this study. These two lines are in the category of Japanese cultivated radish (*Raphanus sativus var. hortensis*) with a long white root. UC-G and MS-G have normal cytoplasm and Ogura-type cytoplasm, respectively. The MS-G was developed by introducing Ogura-type male-sterile cytoplasm into UC-G and maintained by repeated backcrosses.

### Mitochondrial DNA extraction

Mitochondrial DNA was isolated from leaves of MS-G and UC-G, according to the method of Bonen and Gray [47] using discontinuous (1.15 M, 1.30 M and 1.45 M sucrose) density gradient centrifugation with minor modifications. DNaseI-treated mitochondria were collected from the interface between 1.30 M and 1.45 M sucrose. MtDNA was purified by EtBr/CsCl centrifugation and ethanol precipitation.

### Sequence assembly

#### For normal-type mitochondrial genome

Pyrosequencing using the GS-FLX system (Roche) and de novo assembly were conducted by Hokkaido System Science (Sapporo, Japan). A nucleotide sequence of 20,646,046 bp in total was obtained. Sequences were assembled to 105 contigs (4,414 bp long on average). On the basis of the physical map for the normal-type mitochondrial genome, we connected contigs to develop a master circle. If contigs had similarity to plant plastid genome and their depth value was low, we ignored them. All linkages between contigs were confirmed by genomic PCR (Additional file 4 and Additional file 5). By sequencing genomic-PCR product, we determined the sequences between contigs. Sequences were assembled using the software Sequencher ver. 4.9 (Gene Codes Corporation, Ann Arbor, MI, USA), and deposited in a database (DDBJ) under accession No. AB694743.

#### For Ogura-type mitochondrial genome

Pyrosequencing was conducted using the GS-FLX system (Roche) by Takara-Bio (Ohtsu, Japan). A nucleotide sequence of 12,059,770 bp in total for Ogura-type was obtained. Sequences were assembled to 147 contigs (4,402 bp long on average), using GS De Novo Assembler version 2.0 (Roche). The average sequence depth, which was defined as the total nucleotide number used for assembly divided by the total length of contigs, was 36. On the basis of linkage information of the contigs and the physical map of radish mitochondria, we connected contigs to develop a master circle by a parsimonious method, so that each contig appeared at least once to construct the smallest genome [23]. If the depth of linkage was under 10, we did not use the linkages to make the master circle. When it was difficult to judge whether a given contig really appears in the master circle, we checked the existence of the contig in all possible regions by genomic PCR and sequencing (Additional file 6). Sequences were assembled using the software Sequencher ver. 4.9, and deposited in a database (DDBJ) under accession No. AB694744.

### Sequence analysis

The genes encoding known mitochondrial proteins and those for rRNAs, as well as repeated sequences, were identified using the Basic Local Alignment Search Tool (http://blast.ncbi.nlm.nih.gov/Blast.cgi). A tRNA gene search was conducted with the tRNA scan-SE (http://lowelab.ucsc.edu/tRNAscan-SE/) [48]. ORFs encoding hypothetical proteins were identified using Getorf software (http://emboss.dbcls.jp/). ORFs predicted to encode proteins longer than 100 amino acids were picked up in the current analysis.

## Abbreviations

CMS = Cytoplasmic male sterility; mtDNA = Mitochondrial DNA; ORF = Open reading frame; SNP = Single nucleotide polymorphism; SSS = Substoichiometric shifting.

## Competing interests

The authors declare that they have no competing interests.

## Authors’ contributions

YT carried out the sequence data analysis, and drafted the manuscript. MT isolated mitocnodrial DNA from radish. KY participated in the sequence analysis. HY prepared plant materials. TT supervised the work and edited the manuscript. All authors read and approved the final manuscript.

## Supplementary Material

Additional file 1 **List of repeated sequences in normal-type genome.**Repeated sequences(>80 bp, >90% homology) in normal-type genome were listed. Click here for file

Additional file 2 **List of repeated sequences in Ogura-type genome.**Repeated sequences(>80 bp, >90% homology) in Ogura-type genome were listed. Click here for file

Additional file 3 **The sequences homologous to *Brassicaceae* mitochondrial genome plotted on Ogura-type specific region I -III.** A black line indicates the region specific to the Ogura-type mitochondrial genome. Black boxes indicate the sequence homologous to radish and/or other *Brassicaceae* mitochondrial genomes. Click here for file

Additional file 4 **Validation of contig linkage by PCR analysis.** The primer information used for this PCR analysis is described in Additional file 5. Click here for file

Additional file 5 **The primers used for the PCR analysis to validate the linkage between contigs of normal-type genome.** Information on the primers used for the PCR analysis in Additional file 4. Click here for file

Additional file 6 **The primers used for PCR analysis to validate the short repeats.** Information on the primers used for PCR and sequencing, in order to confirm short repeats in Ogura-type genome. Click here for file
