# Supplementary figures and images for: A complete mitochondrial genome sequence of Ogura-type male-sterile cytoplasm and its comparative analysis with that of normal cytoplasm in radish (Raphanus sativus L.)
Source: BMC Genomics. 2012 Jul 31;13:352. doi: 10.1186/1471-2164-13-352 (PMC3473294; doi:10.1186/1471-2164-13-352)

## Slide 1
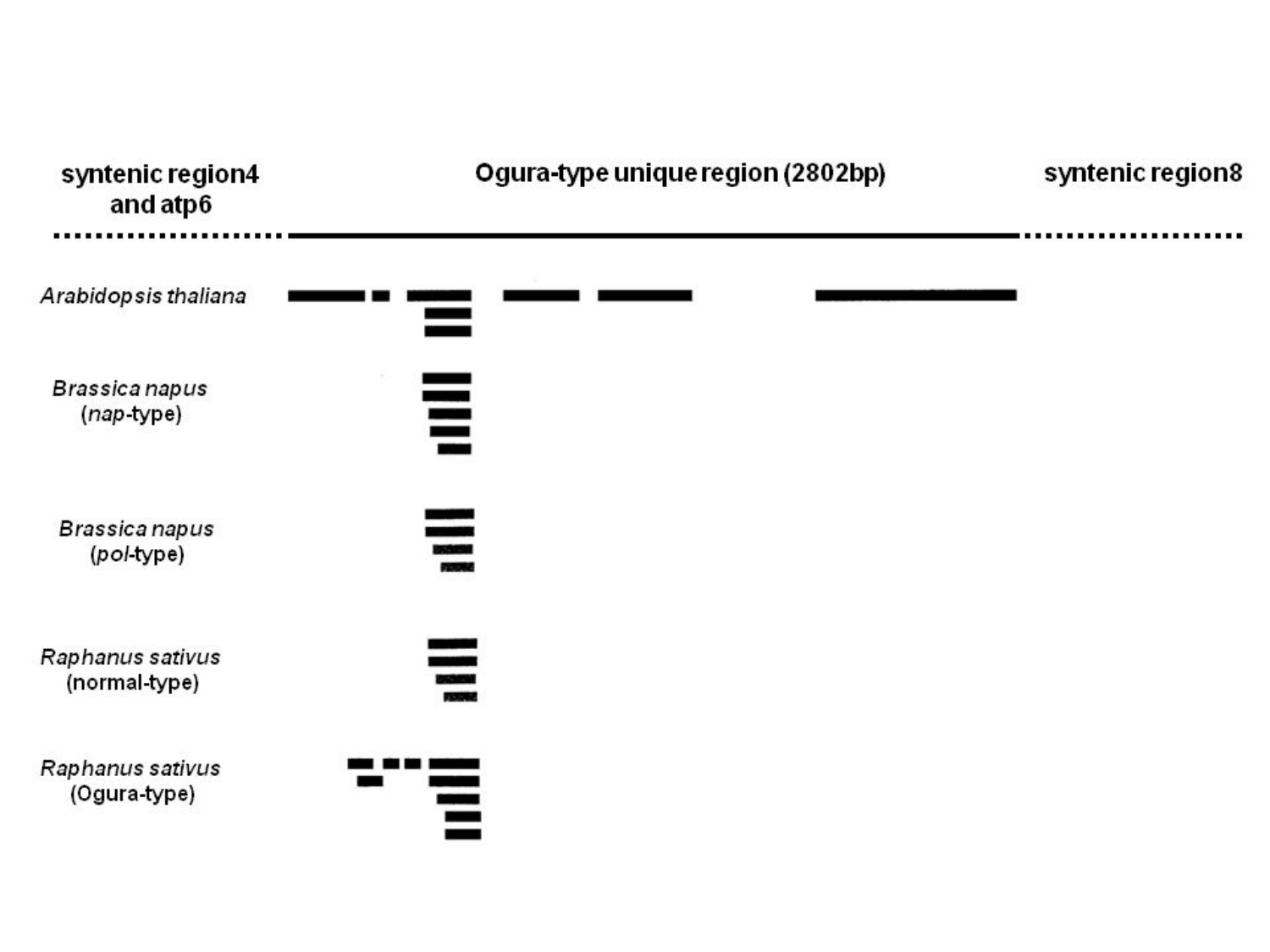

## Slide 2
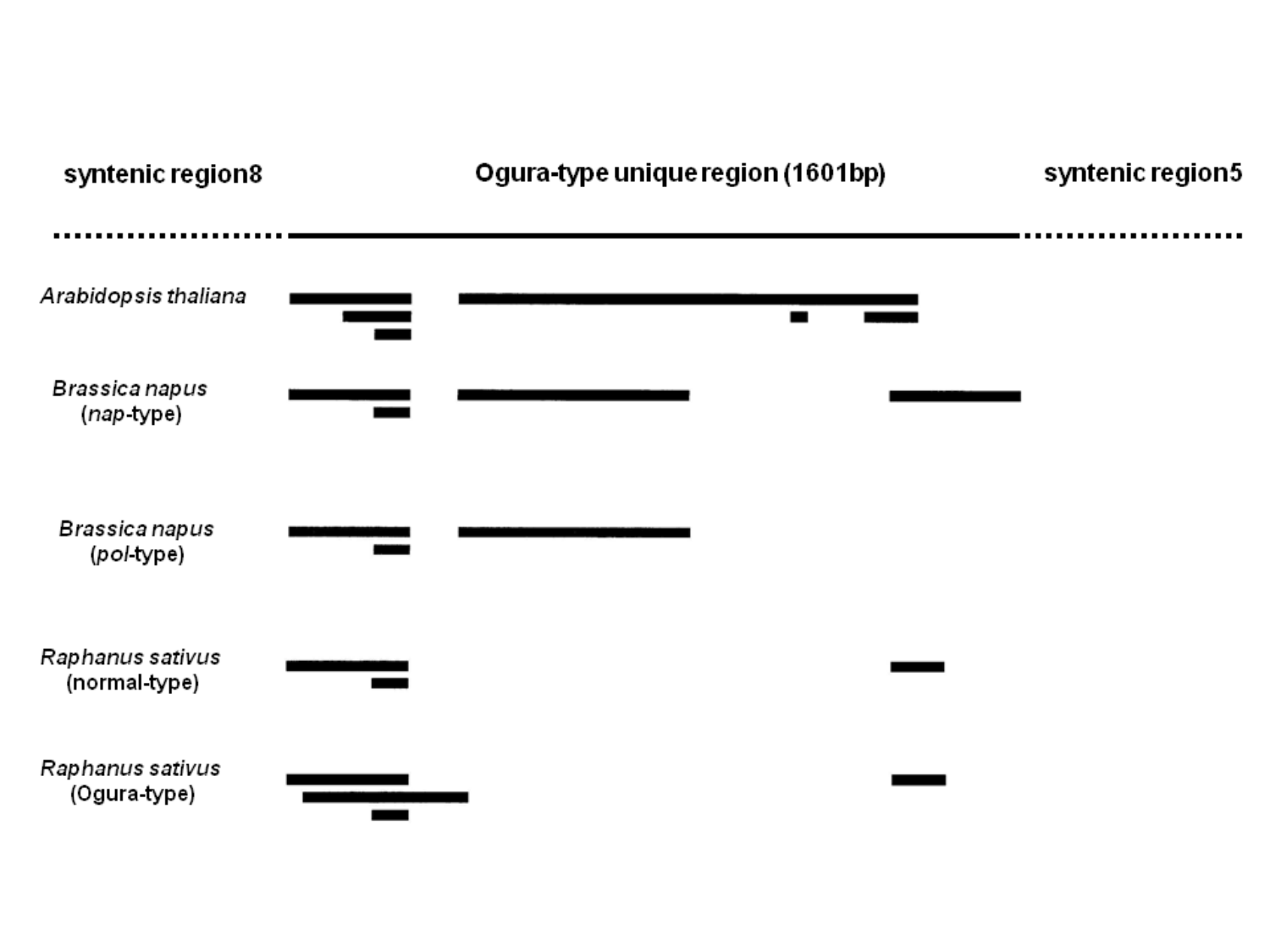

## Slide 3
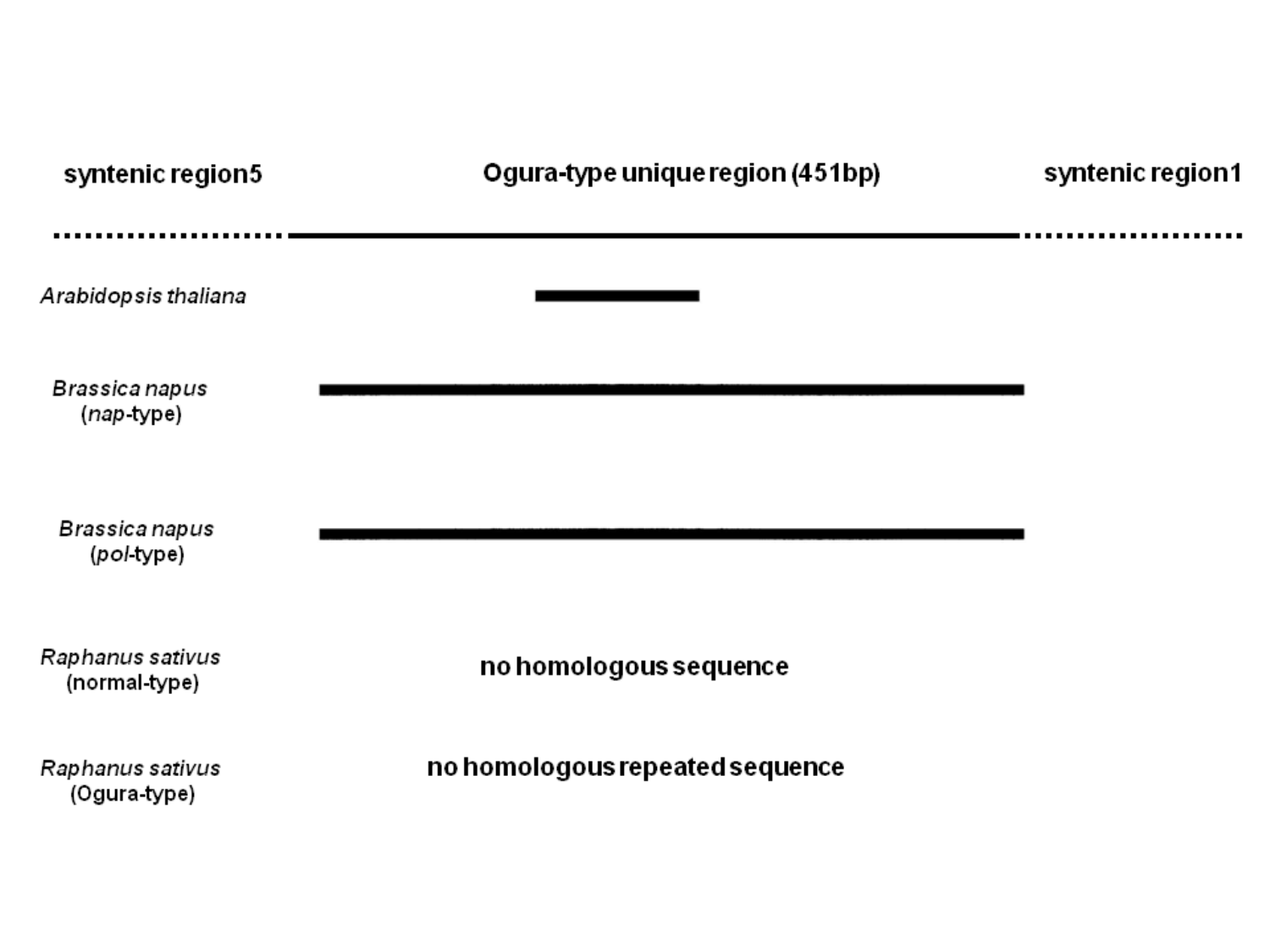

Supplement: Additional file 3 — The sequences homologous to Brassicaceae mitochondrial genome plotted on Ogura-type specific region I -III. A black line indicates the region specific to the Ogura-type mitochondrial genome. Black boxes indicate the sequence homologous to radish and/or other Brassicaceae mitochondrial genomes. [file 1471-2164-13-352-S3.ppt]

## Slide 1
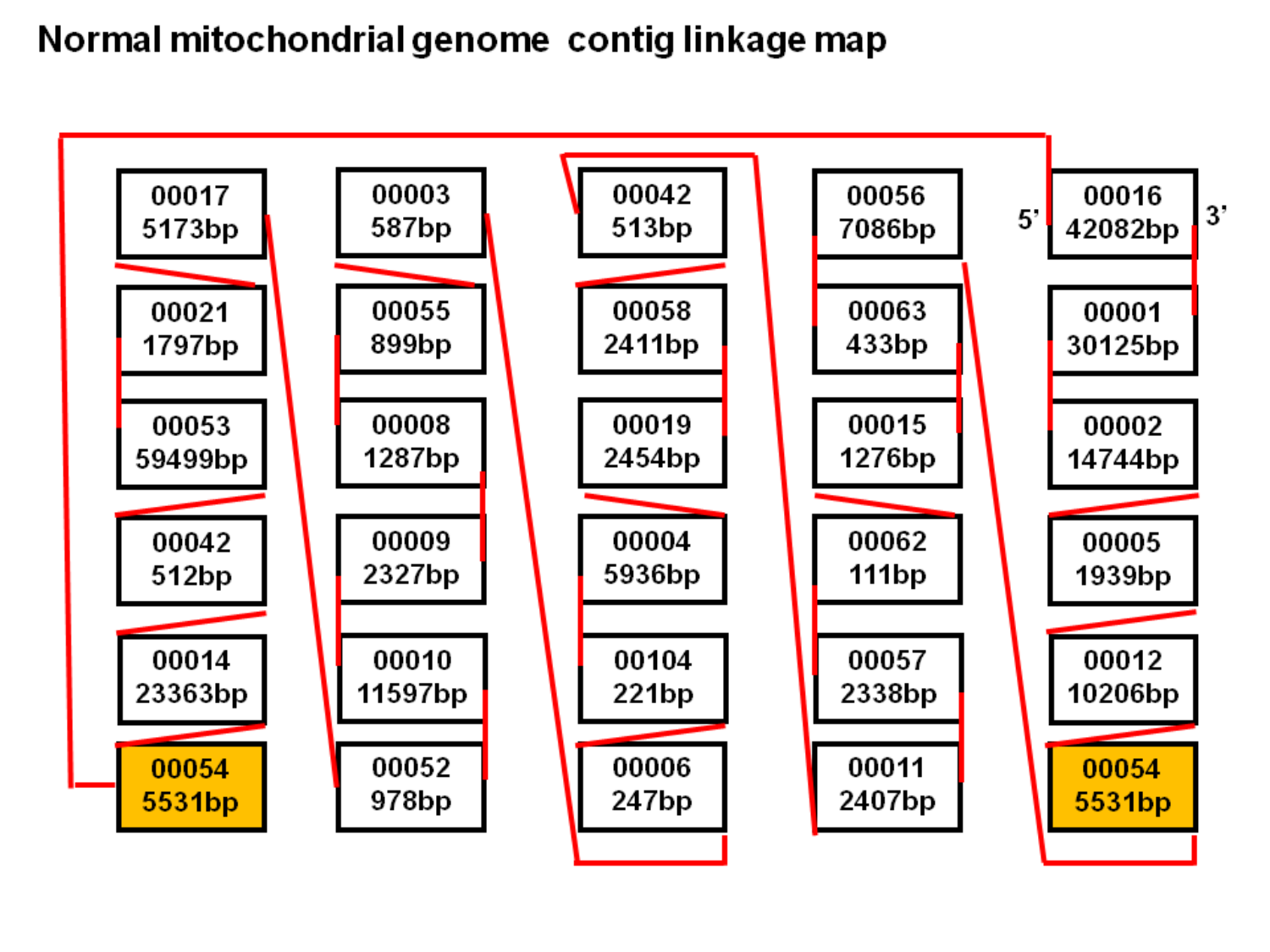

## Slide 2
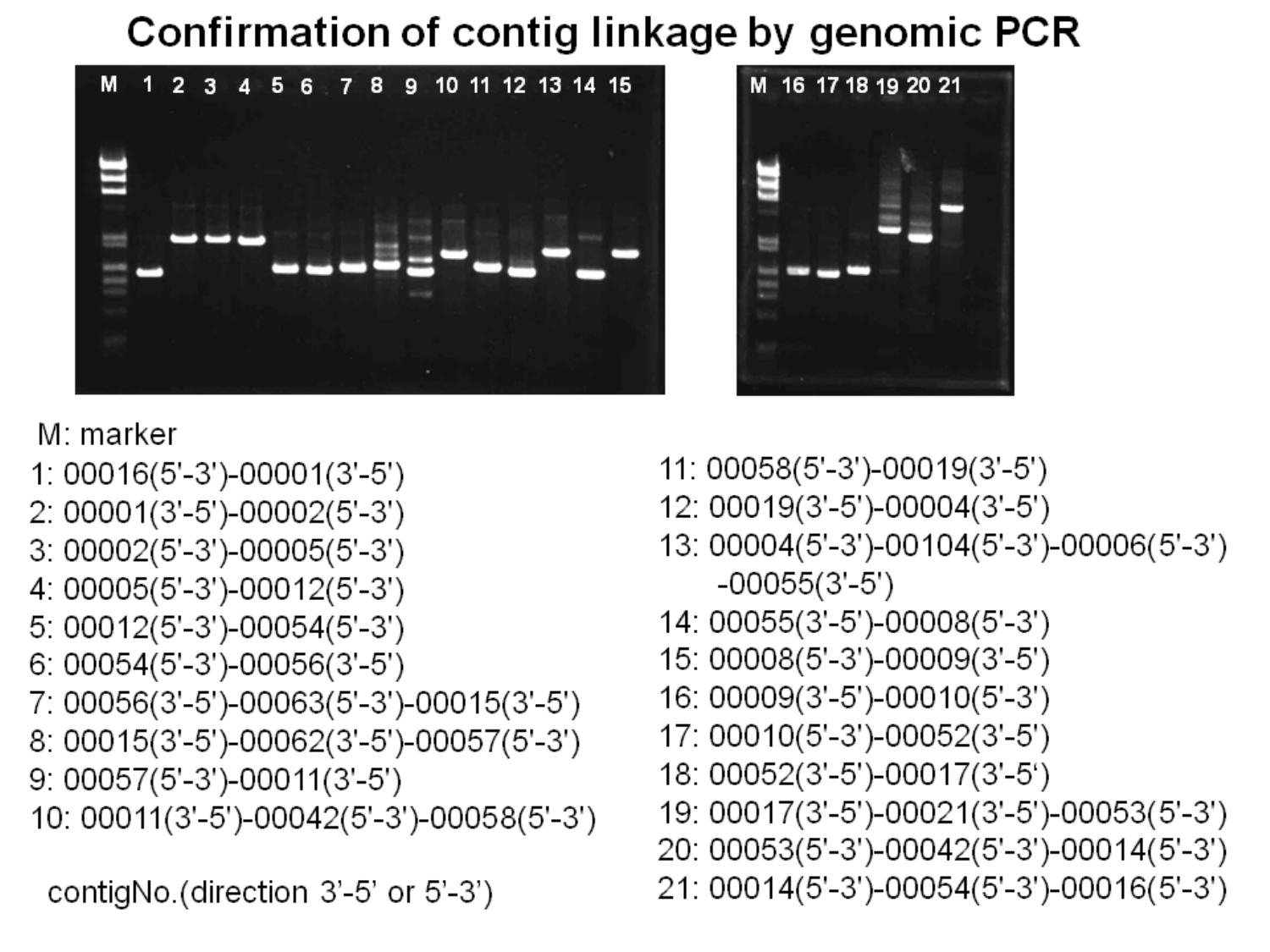

Supplement: Additional file 4 — Validation of contig linkage by PCR analysis. The primer information used for this PCR analysis is described in Additional file 5. [file 1471-2164-13-352-S4.pptx]
